# Emerging Therapeutic Potential of Nanoparticles in Pancreatic Cancer: A Systematic Review of Clinical Trials

**DOI:** 10.3390/biomedicines4030020

**Published:** 2016-08-19

**Authors:** Minnie Au, Theophilus I. Emeto, Jacinta Power, Venkat N. Vangaveti, Hock C. Lai

**Affiliations:** 1 Public Health and Tropical Medicine, College of Public Health, Medical and Veterinary Sciences, James Cook University, James Cook Drive, Douglas, Townsville QLD 4811, Australia; Minnie.au@my.jcu.edu.au; 2 Townsville Cancer Centre, The Townsville Hospital, Townsville QLD 4814, Australia; Jacinta.power@my.jcu.edu.au (J.P.); Hock.Lai@health.qld.gov.au (H.C.L.); 3 College of Medicine and Dentistry, James Cook University, James Cook Drive, Douglas, Townsville QLD 4811, Australia; venkat.vangaveti@jcu.edu.au

**Keywords:** pancreatic cancer, nanoparticles, clinical trials, cancer therapy

## Abstract

Pancreatic cancer is an aggressive disease with a five year survival rate of less than 5%, which is associated with late presentation. In recent years, research into nanomedicine and the use of nanoparticles as therapeutic agents for cancers has increased. This article describes the latest developments in the use of nanoparticles, and evaluates the risks and benefits of nanoparticles as an emerging therapy for pancreatic cancer. The Preferred Reporting Items of Systematic Reviews and Meta-Analyses checklist was used. Studies were extracted by searching the Embase, MEDLINE, SCOPUS, Web of Science, and Cochrane Library databases from inception to 18 March 2016 with no language restrictions. Clinical trials involving the use of nanoparticles as a therapeutic or prognostic option in patients with pancreatic cancer were considered. Selected studies were evaluated using the Jadad score for randomised control trials and the Therapy CA Worksheet for intervention studies. Of the 210 articles found, 10 clinical trials including one randomised control trial and nine phase I/II clinical trials met the inclusion criteria and were analysed. These studies demonstrated that nanoparticles can be used in conjunction with chemotherapeutic agents increasing their efficacy whilst reducing their toxicity. Increased efficacy of treatment with nanoparticles may improve the clinical outcomes and quality of life in patients with pancreatic cancer, although the long-term side effects are yet to be defined. The study registration number is CRD42015020009.

## 1. Introduction

Pancreatic cancer is a rare but aggressive disease that is plagued by a myriad of problems including late diagnosis often when the cancer has metastasised, no early warning symptoms and inadequate therapeutic options on diagnosis [1]. The incidence rate of pancreatic cancer for gender is close to one, with approximate rates of eight per 100,000 in men and six per 100,000 in women globally [2]. Worldwide, it is responsible for 331,000 deaths annually [2]. It is the sixth most common cause of cancer-related death in Australia and the fourth globally [3]. Despite years of research, the five year survival rate remains at approximately 5% [1]. The median age of diagnosis has been reported to range between 66 and 68 years [4]; however, early onset pancreatic cancer occurring in patients under 50 years of age is associated with more advanced disease at presentation and a poorer prognosis [4,5]. Currently 97% of the burden of disease from pancreatic cancer is due to years of life lost to premature death [6] with a median survival time of six to ten months for locally advanced disease, and three to six months for metastatic disease [7,8]. Established risk factors for pancreatic cancer include a family history of the disease and smoking, which account for 5% to 10% of cases. Other weaker associations include obesity, diabetes mellitus, chronic pancreatitis, periodontal disease, *Helicobacter pylori* and gallstones [4]. A challenge to the management of pancreatic cancer is the drug resistant nature of pancreatic tumour cells to gemcitabine, a pyrimidine antagonist used as the first line chemotherapeutic agent [9]. Unlike many other cancers, pancreatic cancer is characterised by several pathophysiological complications that makes it hard to treat, specifically with drugs. Traditionally, complete surgical resection provides the most recognised form of treatment [10]. A complete analysis of the difficulties in treating pancreatic cancer is aptly reviewed by Oberstein and Olive [11].

Nanoparticles are 100 to 10,000 times smaller than human cells and can interact with biological molecules intra and extracellularly [12]. Nanomedicine is the use of nanoparticles in medicine, and they can be attached to lipids or form polymers to encapsulate drugs to increase drug solubility, permeability and delivery to target cells leading to higher therapeutic efficiency [13]. Their unique properties include the ability to remain stable in the physiological environment and passively target pancreatic cancer cells via the enhanced permeability and retention effect (EPR). EPR is due to the size of the nanoparticles, which allow them to extravasate from leaky blood vessels, supplying the carcinoma and targeting it. Due to the poor lymphatic drainage in tumours, nanoparticles are able to accumulate within tumour capillaries and are large enough to escape filtration by the kidney and small enough to evade phagocytic removal by Kupffer cells and splenocytes. However, the non-physiological surface chemistry of nanoparticles may cause non-specific cellular targeting and precipitation leading to cell damage [14]. Alternatively, nanoparticles can be used to actively target tumour cells by combination of specific recognition motifs such as antibodies, sugar molecules, etc. within nanomedicine formulations [15]. Evidence suggests that active targeting by nanoparticles is efficient for poorly leaky tumours, whereas passive targeting is better for highly leaky tumours [15].

Toxicity from nanoparticles may occur as a result of composition, size or charge of the nanoparticles [16]. For example, cationic liposomal nanoparticles can interact with the extracellular matrix, serum proteins and lipoproteins, with consequent aggregation and or oxidative stress resulting in non-target tissue damage [17,18]. Gold nanoparticles are able to cross the placenta and damage the developing foetus [19]. Gold particles are also implicated in the induction of reactive oxygen formation and the initiation of autoimmunity [20]. Given the large diversity of materials used in the construction of nanoparticles, there is an infinite number of combinations of interactions with a high potential of negative interactions that should be taken into consideration to ensure patient safety [21].

A range of in vitro and in vivo animal studies have shown promising results using a variety of nanoparticles as nanocarriers or in combination with standard chemotherapeutic agents [22,23,24,25,26,27,28,29,30,31,32]. There has been a surge of interest in the use of nanoparticles as therapeutic agents for various cancers in recent years. For example, a number of clinical trials have been conducted using nanoparticles as nanocarriers for a range of solid organ tumours such as colorectal cancer [33], non-small cell lung cancer [34], gastric cancer [35], breast cancer [36] and adenocarcinomas of the oesophagus and gastroesophageal junction [37]. Therefore, the aim of this systematic review is to synthesise available literature on clinical trials performed up to March 2016 on the latest developments in the use of nanoparticles as an emerging therapy for pancreatic cancer.

## 2. Methods Section

### 2.1. Literature Search

This systematic review was performed in accordance to the Preferred Reporting Items of Systematic Reviews and Meta-Analyses (PRISMA) statement [38]. The study protocol can be found on the PROSPERO international prospective register of systematic review (PROSPERO 2015: CRD42015020009). Briefly, a literature search to identify studies investigating the use of nanoparticles in the management of pancreatic cancer was conducted. The Embase (1980), MEDLINE (1966), SCOPUS (1996), Web of Science (1965), and Cochrane Library databases (1992) were searched from inception to March 2016 with no language restrictions. Search terms applied included: “nanoparticles” OR “nanomedicine”, [Title/Abstract] AND “pancreatic cancer management” OR “pancreatic cancer therapy”, AND/OR “clinical trials” OR “clinical studies” OR “human participants”. Titles and abstracts were independently screened by two authors (M.A and J.P) to identify possibly relevant studies. The full texts for articles that appear ambiguous were assessed to determine their suitability for inclusion. Database searches were supplemented by scanning the reference lists of included studies and employing the related articles function in PubMed. Subsequently, the full texts of all potentially eligible studies were evaluated in detail for inclusion by the two authors. Discrepancies were resolved in a consensus meeting between the two authors. If the two authors failed to reach a consensus, a third author (T.I.E.) was involved to make a final decision.

### 2.2. Inclusion/Exclusion Criteria

The studies included in this paper are clinical trials involving human participants diagnosed with pancreatic cancer. Interventions used in the studies must include at least one group of participants being treated with nanoparticles for pancreatic cancer, and the impact of nanoparticle treatment on the outcome of disease progression or overall survival must be measured. Studies excluded were studies not involving human participants, studies evaluating the use of nanoparticles in the imaging/diagnosis of pancreatic cancer and not the treatment, and studies evaluating the use of nanoparticles in patients without pancreatic cancer.

### 2.3. Data Collection

Two investigators (M.A and J.P) extracted data using the aforementioned strategy. Data extracted included specific details about the population, interventions, comparison, outcome (PICO) and study methods of significance to the review question and specific objectives. Authors of eligible studies were contacted where additional information was required. Data were cross-checked in a consensus meeting and again, discrepancies were resolved through discussion and mutual agreement between the two authors. The third author (T.I.E.) was available to make a final decision if required.

### 2.4. Quality of Methods Assessment

Two independent reviewers (M.A and J.P) assessed the validity of the studies using the Jadad score [39] for randomised control trials (RCT) and the Therapy CA Worksheet [40], for intervention studies. If there is any disagreement, the third reviewer (T.I.E.) interceded to make a final decision. The Jadad score assesses randomisation, blinding, and attrition to derive a score ranging from 0 (low quality) to 5 (high quality). For this review, a Jadad score greater than 2 was deemed to be of sound methodology. The Therapy CA Worksheet assesses whether the study was randomised, whether there was sufficient and complete follow up, and whether groups were analysed according to their random allocations, blinding, group characteristics and outcome (mean survival). Articles were categorised as “low”, “moderate”, or “high” according to analysis.

## 3. Results

### 3.1. Study Selection

**Figure 1:**
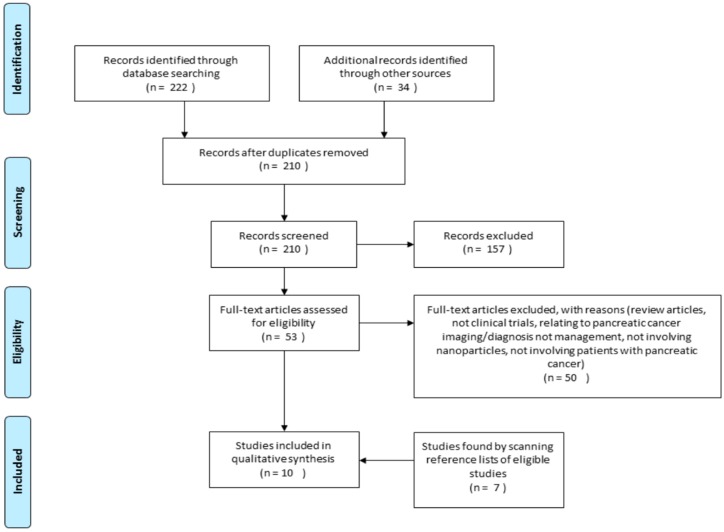
Flow diagram illustrating data collection protocol employed in this study.

We identified 210 potentially eligible studies from initial database searches after removing duplicates (Figure 1). A total of 157 articles were excluded following review of their titles and abstract. The most frequent reasons for exclusion were: not being clinical trials, not involving nanoparticles, and not involving patients with pancreatic cancer. After appraising 53 full text articles, a further 50 were excluded because they were not clinical trials or involved the diagnosis or investigation of pancreatic cancer but not the management. Six additional studies met the inclusion criteria on hand searching the reference lists of included studies; therefore 10 studies were included in this study (Table 1). Ten clinical trials were found from the search strategy, including one randomised controlled trial and nine phase I/II clinical trials. The types of nanoparticles evaluated include nanoparticles containing a retroviral gene, gold nanoparticles, micelle nanoparticles, liposomal nanoparticles and albumin nanoparticles conjugated with chemotherapeutics [7,8,41,42,43,44,45,46,47,48]. In addition to evaluating the effects of the drug on the progression of pancreatic cancer, the maximum tolerated dose and adverse effects were also investigated.

### 3.2. Study Characteristics

**Figure 2:**
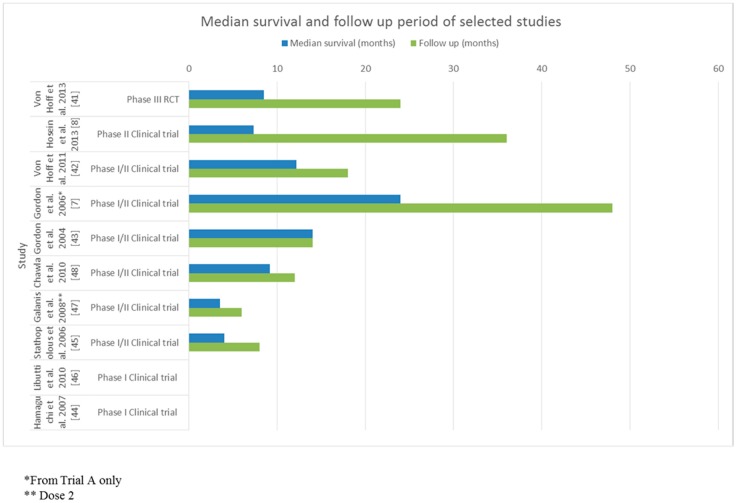
Median survivals and follow up period of selected studies. Abbreviations; RCT = randomised controlled clinical trial.

All studies were prospectively performed, and conducted in hospitals, mostly in tertiary centres in the United States, Australia, Greece and Europe. Three studies (30%) were conducted in Japan and the Philippines [7,43,44]. All studies stated that informed consent was obtained by the participants and were granted ethics approval. All participants had a formal diagnosis of pancreatic cancer confirmed by histology, imaging and tumour markers; the majority had metastatic disease refractory to conventional chemotherapy. The sample size of studies included ranged from 1 to 861 with a median sample size of 12 participants (interquartile range (IQR), 3–23). Participant age was not stated in the two studies, in the rest of the studies, participant age ranged from 27 to 88 years (Table 1). The median survival for participants ranged from 3.5 to 24 months, with an overall median of 8.9 months (IQR, 3.5–13.6), adverse effects ranged from minor ones such as headaches to major effect such as neutropenia and sepsis (Table 2). Two studies (20%) did not state the median survival time, or the follow-up period [44,46] (see Table 1 and Table 2). For the remaining studies (80%), the follow up period ranged from six to 48 months, with an overall median of 16 months (IQR, 9.0–33.0), (Table 2 and Figure 2).

## 4. Synthesis of Study Results

### 4.1. Nanoparticle Albumin Bound Paclitaxel

Paclitaxel is a plant chemotherapeutic alkaloid that is mixed with human serum albumin in an aqueous solvent and is under high pressure to form a 100–200 nm drug nanoparticle albumin bound paclitaxel (nab-paclitaxel) [49]. One phase I/II study and one phase II study were found on investigating the effect of nab-paclitaxel [8,42]. Promising beneficial effects of a combination of nab-paclitaxel and gemcitabine were reported in the first study [42]. The second study involved patients with advanced pancreatic cancers, and failed to show convincing therapeutic effect of this medication [8]. In a phase I/II study involving 67 patients randomised into three groups, 20 receiving 100 mg/m^2^, 44 receiving 125 mg/m^2^ and three receiving 150 mg/m^2^ of nab-paclitaxel, followed by 1000 mg of gemcitabine on three days in every 28 day cycle. Von Hoff and colleagues reported that the maximum tolerated dose of nab-paclitaxel was 125 mg/m^2^ once a week for three weeks plus 1000 mg/m^2^ gemcitabine every 28 days [42]. They found that the dose limiting adverse reactions were neutropaenia and sepsis, the progression free survival was 7.9 months (95% CI 5.8–11 months) with a median overall survival of 12.2 months (95% CI 9.8–17.9 months) and a one-year survival rate of 48%. Positron emission tomography (PET) analysis of patients showed a median decrease in metabolic activity of 79% in all three treatment groups with a higher reduction in metabolic activity in the group receiving 125 mg/m^2^ nab-paclitaxel-gemcitabine compared to those receiving 100 mg/m^2^, 68% vs. 53%, respectively (*p* = 0.044). However, in 19 patients with stage III/IV pancreatic cancer who progressed on gemcitabine-based treatment, and recruited into a single-arm, open-label phase II clinical trial of nab-paclitaxel, Hosein and colleague reported similar side effects to the Van Hoff study above, a progression free survival of 1.7 months (95% CI, 1.5–3.5 months), good overall tolerance, and median overall survival of 7.3 months (95% CI, 2.8–15.8 months) [8].

In a more recent phase III RCT involving 861 participants with metastatic pancreatic cancer randomly assigned to a treatment regimen involving nab-paclitaxel and gemcitabine or gemcitabine alone, the same authors reported a significant increase in median overall survival in the group receiving nab-paclitaxel compared to the group receiving gemcitabine alone of 8.5 months and 6.7 months, respectively (*p* < 0.001) [41]. At the one-year mark, the survival was 5.5 months in the nab-paclitaxel-gemcitabine group compared to 3.7 months in the gemcitabine group (*p* < 0.001). The adverse events associated with treatment were more prominent in patients receiving nab-paclitaxel plus gemcitabine; these include: neutropaenia (38% in the nab-paclitaxel plus gemcitabine group, 27% in the gemcitabine group), fatigue (17% in the nab-paclitaxel plus gemcitabine group, 7% in the gemcitabine group) or neuropathy (17% in the nab-paclitaxel plus gemcitabine group, 1% in the gemcitabine group). However, the rates of myelosuppression and neuropathy were also increased [41]. Taken together, these studies suggest that nab-paclitaxel may serve as a promising treatment modality in the future.

### 4.2. Pathotrophic Nanoparticle Gene Delivery

Rexin-G is a pathotropic retroviral based nanoparticle/gene delivery vector produced by transient co-transfection of human embryonic kidney 293T cells with the Moloney murine leukaemia virus, and encodes a dominant negative mutant construct of the human *cyclin G1* gene [43]. The first clinical trial using Rexin-G in the treatment of pancreatic cancer in the Philippines was performed by Gordon et al. [43]. They reported tumour stabilisation in doses ranging from 2.7 × 10^10^ to 3 × 10^11^ colony forming units; tumour growth was arrested in three of three patients with no experience of dose limiting toxicity. Two patients were stable five and 14 months from diagnosis, respectively. There were no adverse events such as bone marrow suppression, significant alterations in liver and kidney function, nausea or vomiting, mucositis or hair loss. In a further multicentre/country study, the same performed a series of clinical trials investigating the use of Rexin-G in patients with locally advanced or metastatic pancreatic cancer [7]. Clinical trial A assessed the use of Rexin-G in six patients with pancreatic cancer. Five patients showed a partial response and one had stable disease. Half of the participants had a >30% reduction in tumour size by Response Evaluation Criteria in Solid Tumours (RECIST) or by tumour volume measurement. Progression-free survival ranged between two to nine months with a mean of 3.8 months. The median overall survival of patients treated with Rexin-G from diagnosis was 24 months, whereas that for patients on conventional therapy was 4.4 months. Clinically, all six participants had no associated nausea, vomiting, diarrhoea; mucositis, hair loss or neuropathy, although three participants had symptomatic relief of pain. The only adverse reactions association with treatment were a generalised rash and urticaria in two participants [7].

Clinical trial B investigated the effectiveness of Rexin-G in patients with metastatic cancer, and this involved three patients with metastatic pancreatic cancer. For the patients with metastatic pancreatic cancer, two had a partial response with a >30% reduction in tumour size, necrosis of the primary tumour and decrease in number and size of metastatic nodules. One patient had progressive disease. All three had symptomatic relief of pain. These patients did not suffer from any treatment related adverse reactions [7].

Clinical trial C investigated the effectiveness of using a personalised dosing regimen (Calculus of Parity) to calculate the dose of Rexin-G in patients with metastatic cancer. This trial involved two patients with metastatic pancreatic cancer. Both patients responded to therapy with one demonstrating necrosis and cystic conversion of an unresectable pancreatic tumour, whilst the other patient showed significant reduction in the primary pancreatic tumour and a reduction from 28 to 12 pulmonary nodules. None of the patients experienced nausea, vomiting, diarrhoea, mucositis, hair loss or neuropathy. However, two patients developed anaemia requiring packed red cell transfusions, which was potentially due to bleeding into the necrotic tumours [7].

In a similar study involving 13 patients with metastatic pancreatic cancer resistant to standard chemotherapy containing gemcitabine, Chawla et al. reported that four patients left the trial due to complications related to their disease or personal reasons after less than one cycle of therapy [48]. They found that the median overall survival was 2.6 months for six patients at dose level 0–1 (1 × 10^11^ colony forming units, 2–3 times a week) and 9.3 months for seven patients at dose level 2 (2 × 10^11^ colony forming units, thrice a week for four weeks). Treatment related grade 1 adverse events were experienced by three participants; two experienced fatigue and one experienced chills with a headache [48].

Galanis et al. [47] carried out a study to determine the dose of Rexin-G that provided the best response in 12 patients with gemcitabine refractory metastatic pancreatic cancer. The investigators found that at a dose level between 1 × 10^11^ to 6 × 10^11^ colony forming units per cycle, the treatment was mostly well tolerated with only one participant experiencing a dose limiting toxicity of raised serum transaminases at a dose of 1.5 × 10^11^ colony forming units. The median survival was 3.5 months with 11 participants showing progressive disease and one showing radiographically stable disease with clinical deterioration. Although the treatment was well tolerated, there was no evidence of clinical anti-tumour activity; CT and PET scans pre-treatment at day 28 showed significantly increased tumour volume with a mean increase of 204.5% (*p* = 0.001), increase in CA 19.9 by a mean of 204.5% (*p* = 0.001), median increase in PET standardized uptake of fluorodeoxyglucose (FDG) was 36.3% (*p* = 0.0244).

Overall, Rexin-G is reported to selectively targets metastatic cancer sites with associated angiogenesis and increase mean survival in patients with pancreatic cancer.

### 4.3. Gold Nanoparticles

Libutti et al. conducted a clinical trial using CYT-6091 in 30 patients with advanced solid organ cancer, including three participants with pancreatic cancer [46]. CYT-6091 consists of colloid gold nanoparticles with surface bound recombinant tumour necrosis factor and thiolyated polyethylene glycol. They found that CYT9061 selectively targeted tumour tissue in the three patients with pancreatic adenocarcinoma. Electron microscopy examination of biopsies of tumour and adjacent healthy tissue showed that particles in normal tissues were between 0–2 in the three participants with pancreatic adenocarcinoma and 5–6 particles in tumour tissue. There were minor adverse effects reported including lymphopenia, hypoalbuminaemia, electrolyte disturbances and derangement in hepatic enzymes, but did not specify any overall survival [46]. This study suggests that colloid gold nanoparticles combined with recombinant tumour necrosis factor selectively target pancreatic cancer sites, aiding the delivery of chemotherapeutic agents to pancreatic cancer tissue.

### 4.4. Micelle Nanoparticles

Micelle nanoparticles are constructed by using polyethylene glycol as the hydrophilic component and modified polyaspartate as the hydrophobic component which entraps the drug paclitaxel [44]. Paclitaxel is an antimicrotubule chemotherapeutic agent for a range of solid organ cancers; however, its efficacy is limited by poor water solubility [44]. The use of a micelle nanoparticle formulation overcomes this by encapsulating paclitaxel in a “core-shell” that is water soluble and has been shown to have enhanced anti-tumour activity due to the EPR effect [44,50].

Hamaguchi et al. performed a phase I clinical trial to determine the maximum tolerated dose, dose related toxicities, and pharmacokinetics of NK105, a micelle carrier system for paclitaxel [44]. Nineteen cancer patients were recruited, including 11 patients with pancreatic cancer who received IV infusion of NK105. NK105 was generally well tolerated, six patients developed peripheral neuropathy, none of the patients developed clinically significant haematological toxicities. A partial response was seen in a patient with metastatic pancreatic cancer who received 150 mg/m^2^, their liver metastases reduced in size by 90%, although the effect on pancreatic cancer was not specifically reported. Hence, it is unclear whether micelle nanoparticles would be useful in pancreatic cancer management.

### 4.5. Liposomal Nanoparticles

A liposomal-cisplatin nanoparticle (lipoplatin) is constructed from cisplatin and liposomes composed of dipalmitoyl phosphatidyl glycerol, methoxy-polyethylene glycol-distearoyl phosphatidylethanolamine, and soy phosphatidyl choline [51]. Stathopoulos et al. investigated the efficacy and safe dose of lipoplatin with gemcitabine in 24 patients with refractory pancreatic cancer [45]. Response to treatment was determined by CT (computed tomography) measurement of the tumours. A partial response (>50% reduction in the sum of products of the perpendicular diameters of lesions lasting for at least four weeks) was seen in two patients. Stable disease (<50% reduction and <25% increase in the size of the products of two perpendicular diameters of lesions for at least eight weeks) was seen in 14 patients who had. Median survival from the beginning of treatment was four months. The treatment dose of fortnightly administration of up to 100 mg/m^2^ of lipoplatin and 1000 mg/m^2^ of gemcitabine was well tolerated by the participants with no evidence of neurotoxicity or renal toxicity.

### 4.6. Quality of Methods of Included Studies

The quality of methods assessment of 10 studies included is outlined in Table 3. With a Jadad score of 3, the one RCT included is of reasonably sound methodology, (Table 3a). In the other nine non-randomised clinical trials included, the Therapy CA Worksheet indicates that included studies ranged from low to moderate quality of methodology (Table 3b). Common weaknesses identified were: failure to blind, small sample sizes and/ or failure to justify sample size, and failure to identify and account for all confounders.

## 5. Discussion

Pancreatic cancer remain a devastating cause of death globally [3], and is plagued by limited therapeutic options on diagnosis [1]. A significant challenge in the management of pancreatic cancer is the drug resistant nature of first line chemotherapy [9]. The ability of nanoparticles to bypass some of these difficulties due to their unique characteristics has enabled their trials as putative therapeutic agents for pancreatic cancers in recent years. This article appraised available literature on clinical trials performed up to March 2015 on the use of nanoparticles as therapeutic agents for pancreatic cancer.

Overall, clinical trials have demonstrated that nanoparticles can improve the efficacy of anticancer agents [7,8,41,42,43,44,45,46,47,48]. For example, nanoparticles were shown to increase the delivery, cellular targeting of gemcitabine the current first line chemotherapy for pancreatic cancer, whilst reducing associated adverse effects [14]. Gemcitabine is known to be plagued by issues such as low solubility and poor expression of intracellular gemcitabine-uptake regulating nucleoside transporters on pancreatic cells [27]. Additionally, multidrug resistance proteins, the anti-tumour microenvironment such as epithelial-mesenchymal transition cells with migratory and invasive properties, and the hypoxic stroma in pancreatic cancers also play a role as a physical barrier preventing chemotherapeutic agents from targeting pancreatic cancer cells [4]. The evidence reviewed in this article suggest that these barriers are broken by nab-paclitaxel which increases drug bioavailability and delivery to the malignant tissue [31,48,49]. Nab-paclitaxel, for example, has been reported to not only enhance the effect of paclitaxel by increasing its activity and reducing toxicity, but to also acts synergistically with gemcitabine [42]. Since the development of gemcitabine in 1996, eight phase III clinical trials involving chemotherapeutic [52,53,54,55,56,57] or biologic agents [58,59,60] have failed to show an improvement in survival. Improvement was seen in 2006 when a phase III randomised controlled trial demonstrated that erlotinib and gemcitabine lead to an overall survival of 6.42 months which was significantly prolonged compared to gemcitabine and a placebo [61].

Rexin-G was the first targeted genetic medicine reported to show an increase in overall survival with no organ related toxicity [48]. None of the studies reviewed reported any systematic toxicity [7,47,48]. Although one study failed to show any evidence of vector specific- or neutralising antibodies in the sera of the participants, and no evidence of vector DNA integration or recombination events in non-target organs including lymphocytes [48]. Collectively, these studies suggest that Rexin-G is superior to standard chemotherapy in terms of safety profile, efficacy in the management of gemcitabine resistant pancreatic cancer, as well as improving quality of life.

Libutti et al. performed the first clinical trial involving CTY-6091 and reported potential tumour reducing effects with a moderate safety profile [46]. This outcome is supported by previously reported data on the safety of colloid gold in medicine such as in the treatment for rheumatoid arthritis [62]. In support, pre-clinical studies employing CYT-6091 suggest increased accumulation in solid tumours and a reduction in systemic toxicity [63]. Similarly studies using liposomal nanoparticles was reported to exhibit a high safety profile, low toxicity, adequate tumour targeting ability, low immunogenicity and no renal or neurological toxicity [45].

### 5.1. Current Progress

As demonstrated in this systematic review, there is indeed ongoing research into the development of nanotechnology based on the unique tumour microenvironment, which are able to deliver clinically pertinent doses of active formulations to the tumour site while evading various physiological barriers in the fight against pancreatic cancers. Evidence suggests that there is progress in developing nanoparticles able to increase the efficacy per dose of a therapeutic agent by increasing its bioavailability, and that can also be modified for targeted specificity toward cancer cells with negligible damage to non-target tissues which is generally associated with current chemotherapy [7,41,43,44,45,46,47,48,64,65,66,67]. With the establishment of the Alliance for Nanotechnology in Cancer responsible for fostering innovation and collaboration among researchers to expedite the use of nanotechnology for cancer diagnosis and therapy by the United States National Cancer Institute in September 2004. There have been some success in the design and synthesis of nanoparticles that can encapsulate and deliver a diverse suite of cancer targeting therapeutic formulations such as nanoparticles delivering chemotherapy drugs or RNA interference inhibitors [68,69,70,71], and nanoparticles co-delivering two chemotherapeutic drugs at a fraction of the dose with minimal side effects and with the potential to reduce cost [64,65]. There are many other emerging strategies such as the use of nanoparticles (e.g., magnetic nanoparticles) synergistically to improve photodynamic therapy (use of specific wavelength irradiation to selectively kill cancer cells via oxidative stress and caspase-dependent apoptotic mediated mechanisms) [72,73], and photothermal therapy (use of near-infrared light of longer wavelengths to ablate cancer cells) [74], or both [75], or by employing nanoparticles composed of high atomic numbers such as gold nanoparticles [76], titanium oxide nanotubes [77], or gadolinium-based nanoparticles [78] to enhance radiation therapy.

### 5.2. Limitations

When nanoparticles enter the biological environment, the surface proteins associated with the nanoparticle interact with biological molecules; this interaction depends highly on the composition of proteins on the nanoparticle. Inappropriate surface chemistry of nanoparticles have the potential to cause unwanted reactions, reduction in efficacy and adverse effects [21].

Clinical trials in this study involve participants who have refractory pancreatic cancer; further studies need to be done to ascertain the effects of nanoparticles on patients with less localised pancreatic cancer.

Studies included in this review were heterogeneous precluding a meta-analysis. Variability was identified in the way the dosage of nanoparticles for administration was determined; since dosage is related to toxicity, this may be a confounder in the frequency and severity of side effects found. In order for the studies to be comparable, a standardised form of dosing should be used in future studies.

Nanoparticles can be generated in many forms, and only a few of them have been investigated in clinical trials as demonstrated by this study. Many other nanoparticle types have been investigated in in vivo studies with promising results [64,65]. In the future, it is expected that many more clinical trials will be published on these emerging therapies such as quantum dots, carbon nanotubes, paramagnetic nanoparticles, metallic nanoparticles and silver nanoparticles. Since great diversity exists in the form that nanoparticles can take, this study is only representative of gold nanoparticles, micelle nanoparticles, Rexin-G and liposomal nanoparticles. The nanoparticles used in the clinical trials identified in this study vary greatly among themselves, and the results cannot be generalised to all the forms of nanoparticles available.

Multiple cell lines of origin for pancreatic cancer exist; the trials included in this did not identify the cell line of pancreatic cancer for the participants. This is a limitation, as the types of mutation present in the cell line provides information on the growth characteristics, tumourigenicity and chemosensitivity of the tumour [79]. For example, panc-1 cells have a 5× greater ability to invade compared to BxPC-3 cells and Capan-1 cells have a higher angiogenic potential compared to Panc-1 cells [80,81,82,83]. Although there is limited evidence on the best method to obtain cell line information, further research in this area can enhance the interpretation of results from the use of nanomedicine.

### 5.3. Future Research

These studies highlight the potential of nanoparticles to be used in human participants; the results demonstrate a safe toxicity profile and ability to increase overall survival. Despite promising research showing the efficacy and safety of nanoparticles in in vitro and in vivo studies in animal models, more research is required to determine the clearance mechanisms of nanoparticles and their molecular interactions in human participants [14]. The long-term side effects of using nanoparticles are yet to be defined. More randomised controlled trials are required to determine implications of nanomedicine on the quality of life of patients with pancreatic cancer.

## 6. Conclusions

Clinical trials have been performed involving a retroviral vector, albumin, colloid gold, micelles and liposomes. The clinical trials have demonstrated that nanoparticles can be used in conjunction with chemotherapeutic and other agents increasing their efficacy whilst reducing their toxicity. Increased efficacy of treatment with nanoparticles may improve the clinical outcomes and quality of life in patients with pancreatic cancer, although the long-term side effects of these agents remain unknown.

## Figures and Tables

**Table 1:** Characteristics of included studies.

Country/Region	Sample Size	Age Range (Years)	% Males	Previous Treatment	Follow-Up (Months)	Mortality (%)	Assessment	Reference
North America, Eastern Europe, Australia, Western Europe	*n* = 861	27–88	58	None	24	692 total deaths (80) 333 in the treatment group (77) 359 in the gemcitabine group (83)	Nab-paclitaxel plus gemcitabine vs. gemcitabine monotherapy	Von Hoff et al. 2013 [41]
United States	*n* = 19	24–80	47.4	Chemotherapy (gemcitabine containing regimen) *	36	16 at 16 months (84)	Determine the effectiveness of nab-paclitaxel monotherapy as a second line agent	Hosein et al. 2013 [8]
United States	*n* = 67	30–72	48	None	18	32 at 12 months (48)	Identify the safety and maximum tolerated dose of nab-paclitaxel plus gemcitabine	Von Hoff et al. 2011 [42]
United States Philippines	Trial 1 *n* = 6 Trial 2 *n* = 3 Trial 3 *n* = 1	Trial 1 45–64 Trial 2 53–68 Trial 3 Not stated	Not stated	Chemotherapy (gemcitabine containing regimen)	Trial 1: 13 Trial 2: 6 Trial 3: 6	Trial 1: 6 (100) Trial 2: 1 (33) Trial 3: 1 (100)	Trial 1: Determine the safety of Rexin-G at varying doses Trial 2: Determine the safety of Rexin-G at varying doses Trial 3: Determine the effectiveness of a personal dosing regimen for Rexin-G	Gordon et al. 2006 [7]
Philippines	*n* = 3	47–56	33	Surgical resection, chemotherapy (gemcitabine containing regimen) and external beam radiotherapy	14	1 (33)	Evaluate the safety and efficacy of Rexin-G	Gordon et al. 2004 [43]
United States	*n* = 13	50–83	46	Chemotherapy (gemcitabine containing regimen)	12	13 (87)	Determine the effectiveness and most appropriate dose of Rexin-G	Chawla et al. 2010 [48]
Unites States	*n* = 12	42–71	75	Chemotherapy (gemcitabine containing regimen)	6	11 (92)	Determine the effectiveness and most appropriate dose of Rexin-G	Galanis et al. 2008 [47]
United States	*n* = 3	Not stated	Not stated	Chemotherapy	Not analysed	Not analysed	Evaluate the efficacy and safety of CYT6091	Libutti et al. 2010 [46]
Japan	*n* = 11	43–72	Not stated	Chemotherapy	Not analysed	Not analysed	Determine the maximum tolerated dose, safety and efficacy of NK105	Hamaguchi et al. 2007 [44]
Greece	*n* = 24	47–80	46	Chemotherapy	8	17 (71)	Evaluate the safety and efficacy of lipoplatin	Stathopolous et al. 2006 [45]

* Two patients received non-gemcitabine-based frontline therapy.

**Table 2:** Summary of findings associating nanoparticles with pancreatic cancer.

Authors	Study Design	Nanoparticle Formulation	Selection Criteria	Main Objective	Participants *	Overall Median Survival/Outcome	Adverse Reactions †	Conclusion
Von Hoff et al. [41]	Phase III Randomised control trial	Nanoparticle albumin bound paclitaxel (nab-paclitaxel)	Metastatic pancreatic cancer Karnofsky performance status score of 70+	Evaluate the safety and efficacy of nab-paclitaxel plus gemcitabine vs. gemcitabine monotherapy in patients with metastatic pancreatic cancer	*n* = 861. Age 27–88 years. Metastatic pancreatic cancer	8.5 months (95% CI, 7.89 to 9.53)	Major: neutropaenia Minor: fatigue, nausea, vomiting, anorexia and neuropathy	Increased overall survival. Adverse effects of peripheral neuropathy and myelosuppression increased
Hosein et al. [8]	Phase II clinical trial	Nanoparticle albumin bound paclitaxel (nab-paclitaxel)	Pre-treated advanced pancreatic cancer	Evaluate the safety and efficacy of nab-paclitaxel monotherapy in patients with advanced pancreatic cancer	*n* = 19. Age 22–80 years. Stage III and IV pancreatic cancer	7.3 months (95% CI, 2.8–15.8)	Major: sepsis and neutropaenia Minor: fatigue and neuropathy	-
Von Hoff et al. [42]	Phase I/II clinical trial	Nanoparticle albumin bound paclitaxel (nab-paclitaxel)	Untreated advanced pancreatic cancer	Identify the safety and maximum tolerated dose of nab-paclitaxel plus gemcitabine in patients with untreated advanced pancreatic cancer	*n* = 67 Age 30–72 years	12.2 months (95% CI, 9.8 to 17.9)	Major: sepsis and neutropaenia Minor: fatigue and neuropathy	Increased overall survival. Slightly higher occurrence of febrile neutropaenia (3% vs. 1%)
Gordon et al. [7]	(A) Phase I/II clinical trial	Rexin-G	Trial A: Locally advanced pancreatic cancer	Trial A: Determine the safety of Rexin-G at varying doses in patients with locally advanced pancreatic cancer	Trial A, *n* = 6 Age 45–64 years	Trial A: 24 months. (95% CI, 11.1 to 39.5)	Trial A: nil minor or major side effects	Trial A: Increased overall survival. Symptom relief
	(B) Phase I/II clinical trial	Rexin-G	Trial B: Metastatic cancer	Trial B: Determine the safety of Rexin-G at varying doses in patients with various types of metastatic cancer	Trial B, *n* = 3 Age 53–68 years	Trial B: 9 months. (95% CI, 2.4 to 14.9)	Trial B: nil minor or major side effects	Trial B: Increased overall survival. Symptom relief
	(C) Expanded access clinical trial	Rexin-G	Trial C: Solid organ cancer	Trial C: Determine the effectiveness of a personal dosing regimen for Rexin-G in solid tumours. Nanoparticle: Rexin-G (non-replicating retroviral vector expressing a cytocidal gene)	Trial C, *n* = 1, Age (not stated)	Trial C: Unknown	Trial C: Major anaemia requiring red cell transfusions and sporadic thrombocytopaenia	Trial C: Reduction in size of metastatic lesions
Gordon et al. [43]	Phase I/II clinical trial	Rexin-G	Stage 4 pancreatic cancer	Evaluate the safety and efficacy of Rexin-G in patients with stage 4 pancreatic cancer. Nanoparticle: Rexin-G	*n* = 3 Stage 4 pancreatic cancer	14 months ** (95% CI, −5.8 to 31.8)	Major: nil Minor: nil	Increased overall survival. No adverse events
Chawla et al. [48]	Phase I/II clinical trial	Rexin-G	Gemcitabine resistant metastatic cancer	Determine the effectiveness and most appropriate dose of Rexin-G in patients with gemcitabine resistant metastatic cancer. Nanoparticle: Rexin G	*n* = 13 Age 50–83 years Gemcitabine refractory Metastatic disease	2.6 months at dose 0–1, *n* = 6. 9.3 months at dose 2, *n* = 7	Major: nil Minor: fatigue, chills and headache	Increased overall survival. Low severity of adverse events
Galanis et al. [47]	Phase I/II clinical trial	Rexin-G	Gemcitabine resistant metastatic disease	Determine the effectiveness and most appropriate dose of Rexin-G in patients with gemcitabine resistant metastatic cancer. Nanoparticle: Rexin G	*n* = 12 Age 42–71 years Gemcitabine refractory Metastatic disease	3.5 months from treatment initiation	Major: nil Minor: nausea, fever, diarrhoea, hypermagnesaemia and raised liver enzymes (alanine aminotransferase (ALT), aspartate aminotransferase (AST), alkaline phosphate (ALP))	Significant increase in tumour size. Low severity of adverse events
Libutti et al. [46]	Phase I clinical trial	Colloid gold nanoparticle PEGlycated with recombinant TNF	Solid organ cancer	Evaluate the efficacy and safety of CYT6091 in patients with advanced stage cancer	*n* = 3 with Pancreatic cancer	Not specified	Major: nil Minor: lymphopenia, hypoalbuminaemia, hypokalaemia, hypophosphataemia and deranged liver function tests (bilirubin and AST)	Nanoparticle CYT6091 preferentially targets tumour tissue
Hamaguchi et al. [44]	Phase I clinical trial	NK105 (micelle nanoparticle)	Refractory solid organ cancers	Determine the maximum tolerated dose, safety and efficacy of NK105 in 19 patients with refractory solid organ cancers	*n* = 11 Age 43–72 years (range for all participants)	Not specified. Antitumour response of 1 year for 1 patient, one had stable disease for 4 weeks	Major: neutropaenia Minor: fever. Nausea, fatigue, stomatitis, rash, alopecia (for all participants with a solid organ cancer)	Decrease in size of metastatic lesions. Low severity of adverse events
Stathopolous et al. [45]	Phase I/II clinical trial	Lipoplatin	Refractory pancreatic cancer	Evaluate the safety and efficacy of lipoplatin and gemcitabine in patients with refractory pancreatic cancer	*n* = 24 Age 47–80 years. Refractory pancreatic cancer	4 months from beginning of treatment. (Range 2–8 months)	Major: no neurological/renal toxicity Minor: self- resolving abdominal pain. Myelotoxicity (grade 3)	Treatment resulted in symptom relief and a partial response/stable disease. Low severity of adverse events

* = only includes participants with pancreatic cancer. ** = including 1 patientt still alive after 20 months. † Major reactions include clinically significant neurotoxicity, haemotoxicity and renal/liver toxicity. Minor reactions include non-life threatening symptoms that resolve with minimal or no intervention.

**Table 3:** Quality assessment of included studies.

**Table 3a.** Quality assessment of included randomised controlled trial using the JADAD score.												
**Author and Year**	**Randomisation**			**Blinding**			**An Account of All Patients**			**Total Score**		
Von Hoff et al. 2013 [41]	2			0			1			3		
**Table 3b.** Quality assessment of included studies using the Therapy CA Worksheet.												
**Author and Year**		**Randomisation**	**Sufficient and Complete Follow-Up**		**Groups Analysed as per Randomisation**	**Blinding**		**Groups Treated Equally Apart from Intervention**	**Groups Have Similar Characteristics at the Start**		**Median Survival (Months)**	**95% CI**
Hosein et al. 2013 [8]		N	Y		N/A	N		N/A	N/A		7.3	2.8–15.8
Von Hoff et al. 2011 [42]		N	Y		N/A	N		N/A	N/A		12.2	9.8–17.9
Gordon et al. 2006 [7]		Trial A: N Trial B: N Trial C: N	Trial A: Y Trial B:Y Trial C: N		Trial A: N/A Trial B: N/A Trial C: N/A	Trial A: N Trial B: N Trial C: N		Trial A: N/A Trial B: N/A Trial C: N/A	Trial A: N/A Trial B: N/A Trial C: N/A		Trial A: 25 Trial B: 9 Trial C: N/A	12.36–38.30 * 3.58–13.76 * N/A
Gordon et al. 2004 [43]		N	Y		N/A	N		N/A	N/A		13	−2.30–28.30 *
Chawla et al. 2010 [48]		N	Y		N/A	N		N/A	N/A		Dose 0–1:4.3 Dose 2:9.2	N/A †
Galanis et al. 2008 [47]		N	Y		N/A	N		N/A	N/A		3.5	2.66–4.34 *
Libutti et al. 2010 [46]		N	N		N/A	N		N/A	N/A		N/A	N/A †
Hamaguchi et al. 2007 [44]		N	N		N/A	N		N/A	N/A		N/A	N/A †
Stathopolous et al. 2006 [45]		N	Y		N/A	N		N/A	N/A		4	3.37–4.63 *

Abbreviations: CI = Confidence interval; Y = Yes; N = No; N/A = Not applicable. * Calculated based on values in paper. † Unable to calculate based on information in paper.

## Abbreviations

The following abbreviations are used in this manuscript:

%	percentage
CI	confidence interval
FDG	fluorodeoxyglucose
IV	intravenous
mg/m^2^	milligram per meter square
nab-paclitaxel	nanoparticle albumin bound-paclitaxel
RCT	randomised control trials
PET	positron emission tomography
